# Cross-Talk and Physiological Role of Jasmonic Acid, Ethylene, and Reactive Oxygen Species in Wound-Induced Phenolic Biosynthesis in Broccoli

**DOI:** 10.3390/plants12071434

**Published:** 2023-03-24

**Authors:** Ana Mariel Torres-Contreras, Vimal Nair, Carolina Senés-Guerrero, Adriana Pacheco, Mauricio González-Agüero, Perla A. Ramos-Parra, Luis Cisneros-Zevallos, Daniel A. Jacobo-Velázquez

**Affiliations:** 1 Tecnologico de Monterrey, Escuela de Ingeniería y Ciencias, Av. Eugenio Garza Sada 2501 Sur, Monterrey C.P. 64849, Nuevo Leon, Mexico; 2 Department of Horticultural Sciences, Texas A & M University, College Station, TX 77843-2133, USA; 3 Tecnologico de Monterrey, Escuela de Ingeniería y Ciencias, Av. General Ramón Corona 2514, Nuevo Mexico, Zapopan C.P. 45138, Jalisco, Mexico; 4 Institute for Agricultural Research, INIA-La Platina, Postharvest Unit, Santa Rosa 11610, Santiago 8831314, Chile; 5 Tecnologico de Monterrey, The Institute for Obesity Research, Av. General Ramón Corona 2514, Nuevo Mexico, Zapopan C.P. 45138, Jalisco, Mexico

**Keywords:** stress-signaling pathways, cross-talk, wounding stress, phenolic biosynthesis, chemical-genetic approach

## Abstract

Wounding induces phenolic biosynthesis in broccoli. However, there is scarce information about the physiological and molecular mechanisms governing this stress response. In the present study, a chemical-genetics approach was used to elucidate the role of reactive oxygen species (ROS), jasmonic acid (JA), and ethylene (ET) as stress-signaling molecules in the wound-induced phenolic biosynthesis in broccoli. Wounding activated the biosynthesis of ET and JA. Likewise, the wound-induced biosynthesis of ET and JA was regulated by ROS. JA activated primary metabolism, whereas the three signaling molecules activated phenylpropanoid metabolism. The signaling molecules inhibited the wound-induced activation of the *hydroxycinnamoyl-CoA quinate hydroxycinnamoyl transferase* (*HQT*) gene, which is involved in caffeoylquinic acids biosynthesis, and the main phenolics accumulated in wounded broccoli, suggesting that an alternative caffeoylquinic biosynthesis pathway is activated in the tissue due to wounding. ROS mediated the biosynthesis of most individual phenolic compounds evaluated. In conclusion, ROS, ET, and JA are essential in activating broccoli’s primary and secondary metabolism, resulting in phenolic accumulation.

## 1. Introduction

Broccoli is an important source of bioactive compounds that prevent chronic degenerative diseases. Among these bioactive compounds, phenolics present in broccoli possess high antioxidant activity [1]. The use of controlled postharvest abiotic stresses, such as wounding stress, has been proposed as a simple and effective strategy to biofortify horticultural crops with phenolic compounds [2,3]. In this context, the application of wounding stress in broccoli induces the biosynthesis and accumulation of phenolic compounds [4,5,6,7,8]. For instance, Villarreal-García et al. [4] reported that the accumulation of 3-*O*-caffeoylquinic acid, 1,2-disinapoylgentiobiose, and 1,2-disinapoyl-2-ferulolylgentiobiose was observed during the storage of broccoli florets. Likewise, Torres-Contreras et al. [4] reported that 5-*O*-caffeoylquinic acid and caffeic acid content increased by 122.4% and 41.6% in broccoli chops and in florets cut into four even pieces, respectively, immediately after wounding stress. Moreover, the authors reported that 3-*O*-caffeoylquinic acid and 5-*O*-caffeoylquinic acid increased by 46.7% and 98.2%, respectively, in broccoli florets after storage. Similarly, Guan et al. [6] evaluated the effect of different cutting styles on the biosynthesis of phenolics and cellular antioxidant capacity in wounded broccoli, and the authors reported that the higher the wounding intensity (shreds > 1/2 florets > florets > heads) the higher the accumulation of phenolics and the cellular antioxidant capacity of the tissue. However, there is little scientific information on the physiological and molecular mechanisms governing this stress response.

According to previous reports, the wound response in plants starts with a primary signal released from the cytoplasm of the wounded cells. This primary signal has been identified as extracellular adenosine triphosphate (eATP) [9,10,11]. Then, eATP binds to adjacent unwounded cells and triggers the wound response through the biosynthesis of reactive oxygen species (ROS). At the same time, other signaling molecules such as ethylene (ET) and jasmonic acid (JA) are produced, also playing a significant role in the wound-induced activation of the primary and secondary metabolism of plants [12,13,14]. Although the role of these signaling molecules in the postharvest wound-induced activation of the primary and secondary metabolism of the plant cells has been previously reported for crops such as carrots [12,13], their role in the biosynthesis of phenolics in crops such as broccoli has been overlooked.

The use of small chemical compounds that inhibit stress-signaling pathways allows for the application of chemical-genetics approaches to study stress responses [15]. For instance, phenidone (PHEN) and diphenyleneiodonium chloride (DPI) are used as inhibitors of JA and ROS biosynthesis, respectively, whereas 1-methylcyclopropene (1-MCP) blocks ET action [13,14,16,17,18,19,20]. 

The objective of the present study was to evaluate the physiological role of JA, ET, and ROS on activating genes related to the biosynthesis of stress-signaling molecules, primary metabolites, and phenolics. Likewise, the effect of the stress-signaling molecules on the accumulation of individual phenolics was determined. 

## 2. Results and Discussion

A chemical-genetics approach was followed to better understand the role of ROS, ET, and JA on the wound-induced activation of broccoli’s primary and secondary metabolism, where DPI, 1-MCP, and PHEN were used as inhibitors of ROS, ET, and JA, respectively. The expression of genes related to the biosynthesis of signaling molecules, primary metabolites, and phenolics was evaluated in broccoli treated with inhibitors. Likewise, the accumulation of individual phenolics was quantified in the tissue under treatment. 

The concentration of inhibitors applied (PHEN, 10 mM; 1-MCP, 2000 ppb; DPI, 317 μM) and the sampling time (21 h) were determined according to previous studies [5,14,21]. Likewise, the sampling time to evaluate the expression of genes related to the stress-signaling molecules’ biosynthesis (1 h), and primary and secondary metabolite production (9 h) was selected based on a previously reported transcriptome analysis that determined the differential expression of primary and secondary metabolism-related genes as early and late wound responses in broccoli [21]. 

As indicated in our previous reports using a chemical-genetics approach to evaluate the role of signaling molecules on the wound response in plants, two controls were used to determine the effect of inhibitors on the expression of genes and the accumulation of phenolics: chopped broccoli exposed to air and chopped broccoli dipped in distilled water [10,12,14]. Chopped broccoli exposed to air was used as the control for 1-MCP-treated samples, whereas chopped broccoli dipped in water was used as the control for samples treated with PHEN or DPI, either applied alone or in combination with each other.

### 2.1. Role of ROS, ET, and JA Inhibitors on the Wound-Induced Activation of Stress-Signaling Molecules and Primary Metabolites Biosynthetic Genes 

#### 2.1.1. Stress Signaling Molecules’ Biosynthetic Genes

**Figure 1:**
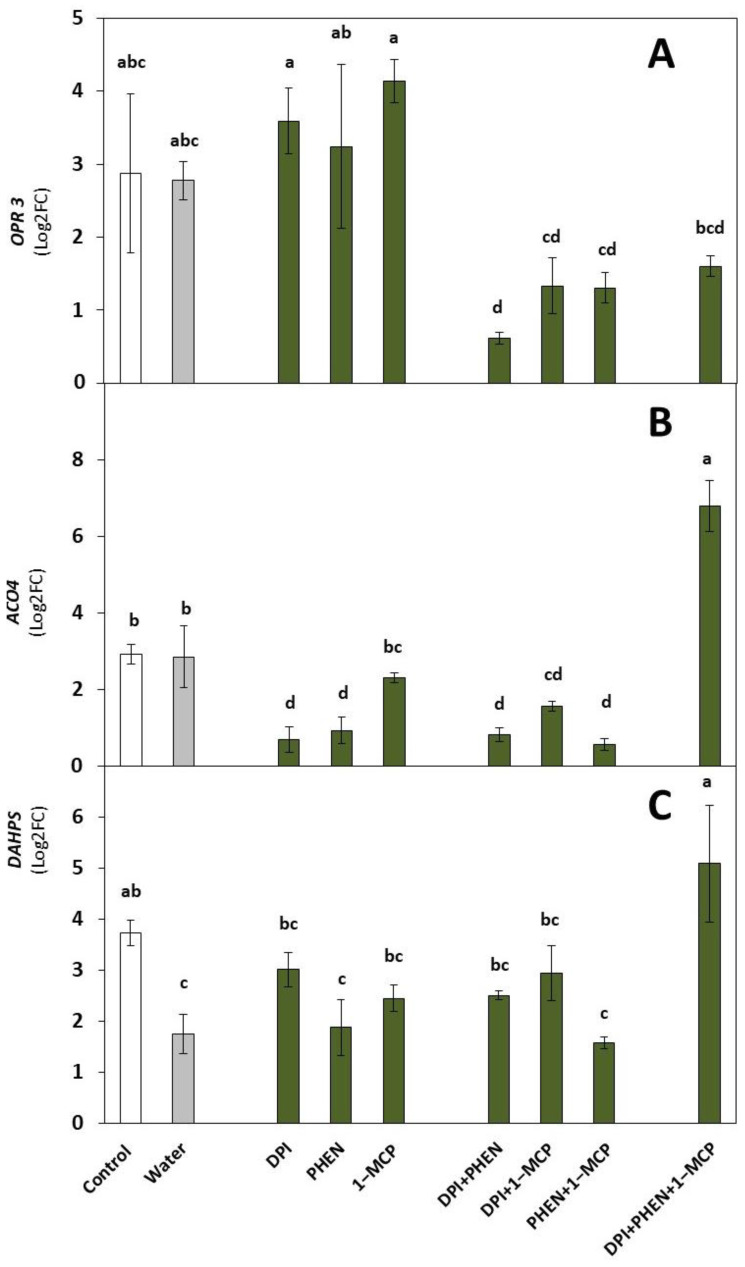
Effect of diphenyleneiodonium chloride (DPI), phenidone (PHEN), and 1-methylcyclopropene (1-MCP) on the relative expression of genes related to the biosynthesis of stress-signaling molecules and primary metabolites. *12-oxophytodienoate reductase 3* (*OPR3*, (**A**)); *1-aminocyclopropane-1-carboxylate oxidase* (*ACO4*, (**B**)); *3-deoxy-D-arabino-heptulosonate synthase* (*DAHPS*, (**C**)). Relative expression is shown at 1 h after wounding. Data represent the mean of 3 replicates ± standard error of the mean. Bars with different letters indicate statistical differences according to the LSD test (*p* < 0.05).

Concerning the evaluation of genes related to the biosynthesis of stress-signaling molecules, the expression of *OPR3* and *ACO4* genes was evaluated (Figure 1). Both genes were wound-induced, in accordance with previous reports [7,21,22]. *OPR3* codes for 12-oxophytodienoate-10,11-reductase (OPR), a key enzyme in JA biosynthesis [22]. The wound-induced activation of *OPR3* was only affected by the combined application of DPI with PHEN (Figure 1A), indicating that ROS and PHEN act together to activate the gene by wounding.

On the other hand, the *ACO4* gene codes for 1-aminocyclopropane-1-carboxylic acid oxidase, which catalyzes a key step in ethylene biosynthesis [23]. *ACO4* showed a significant decrease in its relative expression when DPI and PHEN were applied alone or combined with 1-MCP, indicating that ROS and JA play a vital role in the wound-induced activation of *ACO4* (Figure 1B). Furthermore, when the three inhibitors were applied together, the gene showed higher expression than the control. These results suggest that there is a transcriptional repressor (i.e., ethylene-responsive element binding factors (ERFs)) activated in the presence of the three signaling molecules that modulates the wound-induced production of ethylene [24,25]. 

#### 2.1.2. Primary Metabolites Biosynthetic Gene 

Regarding primary metabolism, the expression of the *DAHPS* gene is shown in Figure 1C. This gene codes for 3-deoxy-D-arabino-heptulosonate synthase, which catalyzes the first step in the shikimate pathway. The wound-induced activation of *DAHPS* was negatively affected by dipping the chops in water. This result indicates that dipping broccoli in water partially removes the wound signal responsible for the gene’s activation. For instance, it has been reported that ATP released from the site of wounding (further referred to as extracellular ATP (eATP)) is the primary signal that induces the wound response in *Arabidopsis* and carrots [9,11].

The individual application of signaling-molecule inhibitors did not significantly reduce the wound-induced expression of *DAHPS* compared with the controls (Figure 1C). However, when the three inhibitors were applied together, the gene showed higher expression than the control. As observed for *ACO4,* these results suggest that there is a transcriptional repressor activated in the presence of the three signaling molecules [24,25]. 

### 2.2. Role of ROS, ET, and JA Inhibitors on the Wound-Induced Activation of Phenolic Biosynthetic Genes and Individual Phenolic Accumulation 

#### 2.2.1. Phenolic Biosynthetic Genes

**Figure 2:**
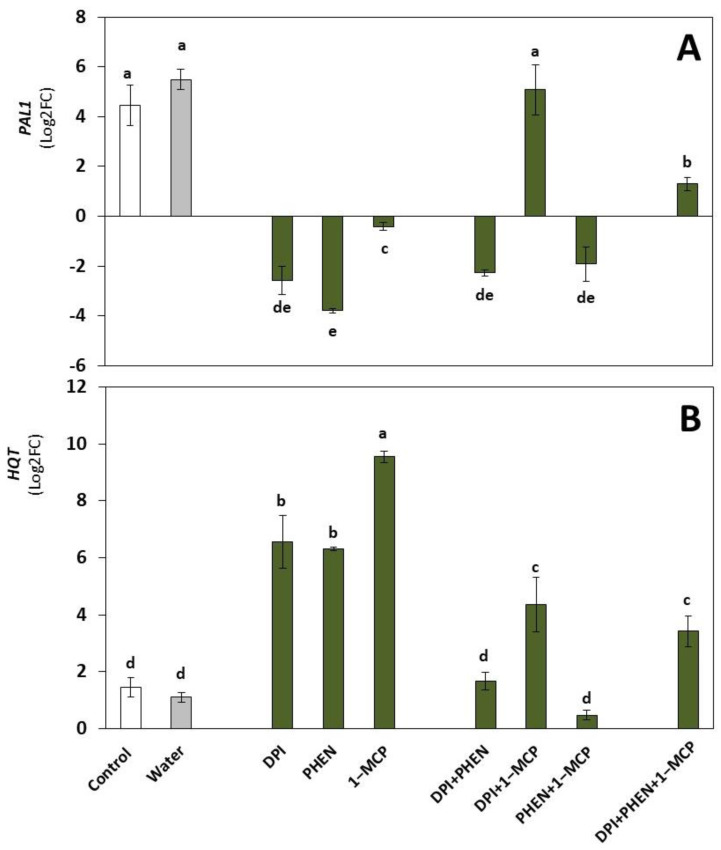
Effect of diphenyleneiodonium chloride (DPI), phenidone (PHEN), and 1-methylcyclopropene (1-MCP) on the relative expression of phenolic biosynthetic genes. Phenylalanine ammonia-lyase (*PAL1*, (**A**)); hydroxycinnamoyl-CoA quinate hydroxycinnamoyl transferase (*HQT*, (**B**)). Relative expression is shown at 9 h after wounding. Data represent the mean of 3 replicates ± standard error of the mean. Bars with different letters indicate statistical differences according to the LSD test (*p* ≤ 0.05).

Concerning phenolic biosynthesis-related genes, the *phenylalanine ammonia-lyase 1* (*PAL1*) and *hydroxycinnamoyl-CoA quinate hydroxycinnamoyl transferase* (*HQT*) were evaluated. PAL catalyzes the first step in the phenylpropanoid pathway and thus is a key enzyme in the biosynthesis of phenolic compounds, while *HQT* is a gene that codes for a key enzyme in the biosynthesis of caffeoylquinic acids [26,27]. Both genes were activated by wounding stress (Figure 2). This result is in accordance with previous reports where *PAL* and *HQT* were induced by wounding in broccoli [7,21].

The application of each inhibitor produced the repression of the wound-induced expression of *PAL1* and down-regulated its expression compared with the control (Figure 2A). Interestingly, the application of DPI in combination with 1-MCP did not affect the wound-induced activation of *PAL1*, suggesting that ROS and ET in conjunction induce the expression of a transcriptional repressor that regulates the wound-induced expression of *PAL1*. On the other hand, the relative expression of *HQT* increased when using the inhibitors individually (Figure 2B). This result suggests that the three signaling molecules (ROS, JA, or ET) down-regulate the expression of *HQT*. When the inhibitors were combined, lower activation of the *HQT* gene was observed as compared with the inhibitors applied alone, indicating a cross-talk between the signaling molecules regulating the gene’s expression. These results are in contrast with a previous report where the application of different plant hormones, including MeJA, induced the activation of *HQT* in potatoes [28]. 

#### 2.2.2. Identification of Individual Phenolic Compounds

**Figure 3:**
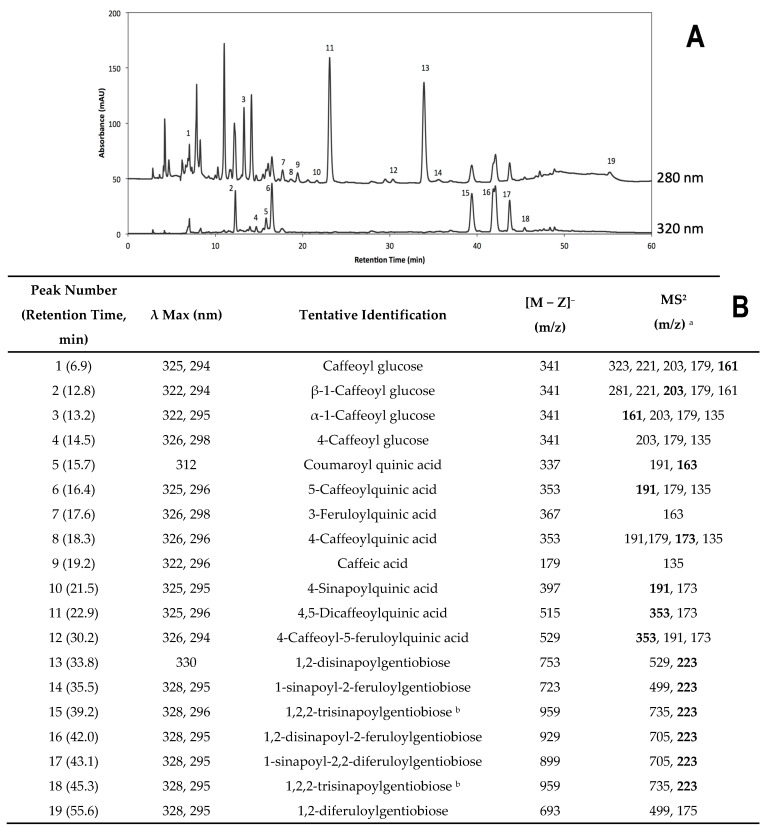
Typical HPLC-DAD chromatogram of phenolic compounds ((**A**), shown at 280 and 320 nm) obtained from methanol/water (70/30, *v*/*v*) extracts in broccoli. Identification of individual phenolics was achieved using HPLC-DAD and HPLC-ESI-MSn (**B**). ^a^ Major fragmentations are highlighted in bold. ^b^ Isomeric compounds.

Individual phenolic compounds identified included hydroxycinnamic acid derivatives: caffeoyl glucose (C-glu); β-1-caffeoyl glucose (β-1-C-glu); α-1-caffeoyl glucose (α-1-C-glu); 4-caffeoyl glucose (4-C-glu); coumaroylquinic acid (Coumaroyl-QA); 5-caffeoylquinic acid (5-CQA); 3-feruloylquinic acid (3-FQA); 4-caffeoylquinic acid (4-CQA); caffeic acid (CA); 4-sinapoylquinic acid (4-SQA); 4,5-dicaffeoylquinic acid (4,5-diCQA); 4-caffeoyl-5-feruloylquinic acid (4-C-5-FQA); sinapic acid derivatives: 1,2-disinapoylgentiobiose (1,2-DSG); 1-sinapoyl-2-feruloylgentiobiose (1-S-2-FG); 1,2,2-trisinapoylgentiobiose (1,2,2-TSG); 1,2-disinapoyl-2-feruloylgentiobiose (1,2-DS-2-FG); 1-sinapoyl-2,2-diferuloylgentiobiose (1-S-2,2-diDFG); and a feruloyl acid derivative: 1,2-diferuloylgentiobiose (Figure 3). The phenolic profile identified in the present study is similar to that found in previous reports [4,29].

#### 2.2.3. Accumulation of Individual Phenolic Compounds 

The effect of DPI, PHEN, and 1-MCP on the wound-induced accumulation of individual PC in broccoli is shown in Table 1. The application of wounding stress induced the accumulation of β-1-caffeoyl glucose (β-1-C-glu), α-1-caffeoyl glucose (α-1-C-glu); 4-caffeoyl glucose (4-C-glu), coumaroyl quinic acid (coumaroyl-QA), 5-caffeoylquinic (5-CQA), 3-feruloylquinic acid (3-FQA), 4-caffeoylquinic acid (4-CQA), and an isomeric form of 1,2,2-trisinapoylgentiobiose (1,2,2-TSPG). Moreover, wounding induced the de novo synthesis of 1-sinapoyl-2-feruloylgentiobiose (1-S-2-FG) and 1,2-diferuloylgentiobiose (1,2-DFG); whereas the de novo synthesis of 4-caffeoyl-5-feruloylquinic acid (4-C-5-FQA) and 1,2-diferuloylgentiobiose (1,2-DSG) occurred only in the presence of a specific signaling molecule inhibitor (Table 1). The use of DPI reduced (−23%, *p* < 0.05) the wound-induced accumulation of β-1-C-glu (Table 1), showing that ROS plays an essential role in its accumulation, since the other inhibitors did not cause an effect. In the case of the isomer form α-1-C-glu, 1-MCP induced a 120% increase compared to the control, indicating that ET inhibits its accumulation (Table 1). 

Coumaroyl-QA, which is the precursor of caffeoylquinic acid, showed the most significant wound-induced accumulation among the caffeoylquinic acid derivatives (450%, *p* < 0.05) in both the control and samples dipped in water (Table 1). The use of DPI alone caused the complete inhibition of coumaroyl-QA accumulation, while PHEN and 1-MCP applied individually also decreased its wound-induced accumulation by around 82% and 32%, respectively. The application of 1-MCP in combination with either DPI or PHEN completely inhibited the wound-induced production of coumaroyl-QA, while the application of the three inhibitors only inhibited 35% of the biosynthesis (Table 1). These results correlated with the expression of *HQT* (Figure 2B) in the treatments, where the use of inhibitors individually induced its expression, indicating that coumaroyl-QA was rapidly converted into caffeoylquinic acid, avoiding its accumulation. Therefore, ROS, ET, and JA played a role in the biosynthesis and accumulation of coumaroyl-QA.

Likewise, 5-CQA accumulated (36%) in response to wounding; however, when broccoli was dipped in DPI or PHEN solution, there was no accumulation of the compound, indicating that ROS and JA were necessary for the wound-induced accumulation of 5-CQA. Interestingly, 5-CQA accumulation has been previously associated with increased expression of HQT in different plant tissues [27,28,30]. Here, the increase in the expression of HQT in the presence of inhibitors (Figure 2B) does not correlate with the accumulation of 5-CQA (Table 1). This result suggests that alternative routes, such as the hydroxylation of p-coumaroyl-quinic acid by p-coumarate 3′-hydroxylase (C3H) or the use of caffeoyl-glycoside as the activated intermediate for 5-CQA biosynthesis [28], are favored in broccoli and occur in the tissue under wounding stress. 

Interestingly, the accumulation of the 5-CQA compound occurred when using DPI and PHEN together (Table 1). A similar trend was observed for 3-FQA, in which the accumulation happened in the presence of ROS and JA. These findings agree with a previous study that reported a complex cross-talk between ROS, JA, and ET that induced the accumulation of phenolics due to wounding stress in carrots [12]. The authors proposed that ROS play a key role as signaling molecules in the wound response, while ET and JA were essential to modulate ROS levels.

It is well known that wounding can induce the de novo synthesis of phenolics. In this context, a sinapic acid derivative (1-S-2-FG) and a feruloyl acid derivative (1,2-DFG) were synthesized de novo in response to wounding (Table 1). The production of 1,2-DFG was not affected by inhibitors, while DPI combined with PHEN induced a higher accumulation of 1-S-2-FG (2189 mg/kg DW). This compound is highly relevant from a nutraceutical perspective, since it is one of the compounds with the highest antioxidant activity reported in broccoli and is effective at preventing lipid damage [1].

The biosynthesis of 4-C-5-FQA and 1,2-DSG was induced de novo only by applying a specific inhibitor or a specific combination of them. PHEN alone and 1-MCP in combination with either DPI or PHEN accumulated 4-C-5-FQA. The accumulation of 1,2-DSG was detected with the application of all the combinations of inhibitors, suggesting that the signaling molecules studied (ROS, ET, and JA) inhibit the biosynthesis of this compound.

**Figure 4:**
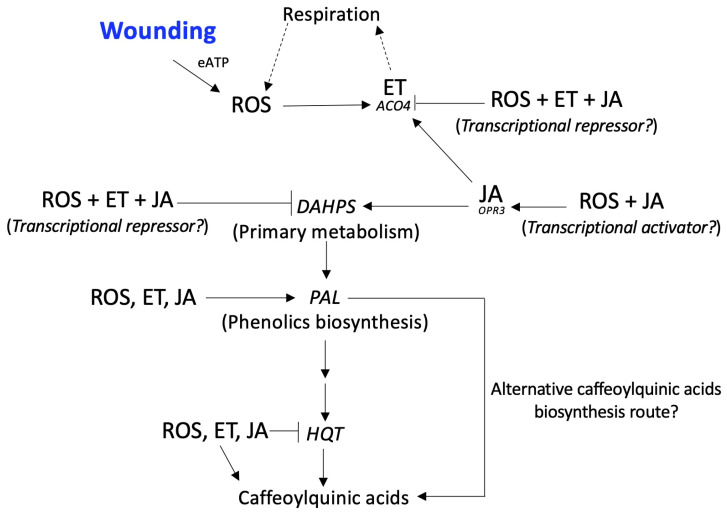
Proposed model explaining the cross-talk and physiological role of reactive oxygen species (ROS), ethylene (ET), and jasmonic acid (JA) in wound-induced phenolic biosynthesis in broccoli. After wounding, extracellular ATP (eATP) was released from the plant cell’s cytoplasm and served as the primary signal of the wound response, triggering ROS production. JA and ROS alone induced ET biosynthesis, whereas we saw the highest expression of the *ACO4* gene when the three signaling molecules were blocked. ROS and JA induced the biosynthesis of JA through the activation of *OPR3*. JA played a major role in the wound-induced activation of *DAHPS*, whereas inhibiting the three signaling molecules increased the expression of the gene, suggesting that the three signaling molecules activated a transcriptional repressor. The three signaling molecules played an important role in the activation of *PAL*. The main phenolic compounds accumulated due to wounding were the caffeoylquinic acids. None of the signaling molecules played a role in the activation of *HQT*, which coded for an enzyme involved in caffeoylquinic acid biosynthesis, suggesting an alternative caffeoylquinic acid biosynthesis route activated by wounding in broccoli.

In summary, wounding stress in broccoli induced the accumulation of phenolics through a complex cross-talk among ROS, ET, and JA. According to the results obtained herein, Figure 4 summarizes the effect of signaling molecules on the activation of genes related to the biosynthesis of signaling molecules, primary metabolites, and phenolics. Previous reports have indicated that upon the application of wounding, eATP is released from the plant cell’s cytoplasm and serves as the primary signal of the wound response, which triggers ROS production [9,10,11]. The application of signaling molecules inhibitors revealed that JA and ROS alone, as well as ROS in combination with ET (likely through a transcriptional activator), induce ET biosynthesis, whereas ET was likely overproduced when the three signaling molecules were blocked, as was indicated by the highest expression of the *ACO4* gene. Regarding jasmonic acid biosynthesis, the results showed that ET alone or the combination of ROS and JA induced the biosynthesis of JA through the activation of *OPR3*.

Regarding the primary metabolism, JA played a major role in the wound-induced activation of *DAHPS*, whereas inhibiting the three signaling molecules increased the expression of the gene, suggesting that there was a transcriptional repressor activated in conjunction by ROS, ET, and JA or that other signaling molecules were being produced (i.e., salicylic acid, abscisic acid, etc.) to compensate for the absence of ROS, ET, and JA. The three signaling molecules played an important role in activating *PAL*, which coded for the key enzyme involved in phenolic biosynthesis. The main phenolic compounds accumulated due to wounding were caffeoylquinic acids. Interestingly, none of the signaling molecules evaluated played a role in the activation of *HQT*, which coded for an enzyme involved in caffeoylquinic acids biosynthesis, the major phenolics accumulated in the wounded tissue. Thus, it was likely that an alternative caffeoylquinic acid biosynthesis route is activated by wounding in broccoli, such as the hydroxylation of p-coumaroyl-quinic acid by p-coumarate 3′-hydroxylase (C3H) or the use of caffeoyl-glycoside as the activated intermediate for 5-CQA biosynthesis [28].

## 3. Materials and Methods

### 3.1. Chemicals

1-methylcyclopropene (1-MCP) powder (SmartFresh^TM^) was obtained from AgroFresh Inc. (Springhouse, PA, USA). Diphenyleneiodonium chloride (DPI), phenidone (PHEN), formic acid, and methanol (HPLC grade) were purchased from Sigma Chemical Co. (St. Louis, MO, USA).

### 3.2. Plant Material, Processing, and Storage of Broccoli Samples

Broccoli (*Brassica oleracea* var. italica) cultivar Heritage was obtained in Monterrey (Nuevo León, México) from a local distributor. Broccoli heads were disinfected with chlorinated water (200 ppm, pH 6.5) and subjected to wounding stress to obtain florets and chops. Florets were obtained using a commercial straight-edged knife, whereas chops were obtained from broccoli florets with a food processor (Waring Commercial, WFP11, Torrington, CT, USA). 

### 3.3. Application of Stress-Signaling Molecules Inhibitors 

Stress signaling molecule inhibitors (1-MCP, 2000 mg L^−1^; PHEN, 10 mM; DPI, 317 μM) were applied individually or in combination in broccoli chops, as previously described [12,13,14]. To determine the effect of inhibitors’ application on the expression of genes and the accumulation of secondary metabolites, two controls were used: chopped broccoli exposed to air (control for 1-MCP treatment) and chopped broccoli dipped in distilled water (control for DPI or PHEN treatments). Samples were stored inside airtight plastic containers in an incubator (VWR, Radnor, PA, USA) at 20 °C for 21 h. Samples were collected during storage to determine the expression of genes as early (1 h) and late responses (9 h) to wounding stress and assess the accumulation of individual phenolics (21 h). After sampling, the tissue was immediately frozen with liquid nitrogen and stored at −80 °C until needed.

### 3.4. RNA Extraction and Quantitative Real-Time Reverse Transcription-PCR (qRT-PCR)

RNA was extracted following the hot borate method [31]. RNA quality and RNA integrity number (RIN) were determined as described by Torres-Contreras et al. [21]. Total RNA was treated with DNAse using RNAse Free DNAse (Qiagen, Hilden, NRW, Germany) and cleaned using the RNeasy Plant Mini kit (Qiagen, Hilden, NRW, Germany). Total RNA quality was determined with the following parameters: OD 260/280 > 1.9 and OD 260/230 > 1.5. Three independent RNA extractions of all samples were performed.

RNA was used to synthesize cDNA with the AffinityScript qPCR cDNA synthesis kit (Agilent Technologies, Santa Clara, CA, USA). Quantification of transcripts generated from cDNA was determined as described by Torres-Contreras et al. [21]. Conditions, procedures, and analysis of qRT-PCR data were performed as described by Salzman et al. [32], using three biological replicates and three technical replicates for each gene validated (*n* = 9). The amplification specificity of each set of primers (Table 2) was determined by analyzing the cleavage curve and amplicon size on agarose gel electrophoresis to ensure the absence of non-specific PCR products. Differential gene expression was calculated using the 2^−ΔΔCt^ method [33].

### 3.5. Phenolic Compounds Analysis

Phenolic compounds were extracted from freeze-dried broccoli (200 mg) by homogenizing the tissue with methanol (5 mL) with further centrifugation (10,000× *g*, 1 h, at 4 °C). The clear supernatant (methanol extract) was filtered using nylon membranes (0.45 μm, VWR) before injection into the chromatographic system. Individual phenolics were identified and quantified by high-performance liquid chromatography (HPLC) with diode-array detection (DAD) and HPLC- electrospray ionization (ESI)-sequential mass spectrometry (ESI-MS*^n^*) with the method reported by Villarreal-García et al. [4]. Briefly, the determination of individual phenolics was performed on a Surveyor HPLC/MS system equipped with an autosampler, a Surveyor 2000 quaternary pump, and a Surveyor UV 2000 PDA detector using a C_18_ reverse phase (150 mm × 4.6 mm, Atlantis, Waters, Ireland; particle size = 5 μm) column connected to an LCQ Deca XP Max MS*^n^* system (Thermo Finnigan, San Jose, CA, USA) with a *Z*-spray ESI source run by Xcalibur software, version 1.3 (Thermo Finnigan-Surveyor, San Jose, CA, USA). The mobile phase flow rate was set at 0.3 mL/min, while the elution gradients were performed with water (phase A) and methanol/water (60:40, *v*/*v*, phase B), both phases in combination with 1% formic acid. The gradient solvent system was 0/100, 4.8/70, 12.8/50, 56/30, 64/20, 72/0, 80/0, and 96/100 (min/% phase A). The chromatograms were monitored at 280, 320, and 360 nm, and complete spectral data were recorded in the 200–600 nm range. ESI was performed in the negative ionization mode, nitrogen was used as a sheath gas with a flow of 60 arbitrary units, and He gas was used as dampening gas. The capillary voltage was 45.7 V, the spray voltage was 1.5 kV, the capillary temperature was 285 °C, and the tube lens voltage was 30 V. Collision energies of 30% were used for the MS*^n^* analysis. A standard curve of 5-caffeoylquinic acid (5-CQA) was prepared to quantify individual phenolics in the range of 0.5–100 mg L^−1^. Individual phenolic concentration was expressed as mg of 5-CQA equivalents per kg of broccoli on a dry weight (DW) basis.

### 3.6. Statistical Analysis

Statistical analyses were performed using three replicates. Data represent the mean values of samples and their standard errors. Analyses of variance (ANOVA) were conducted using JMP software version 9.0 (SAS Institute Inc., Cary, NC, USA), and mean separations were performed using the LSD test (*p* < 0.05). 

## 4. Conclusions

The results from the present study demonstrate that ROS, ET, and JA play an essential role in the wound-induced activation of broccoli’s primary and secondary metabolism, leading to the accumulation of phenolic compounds. JA plays a major role in activating the primary metabolism, whereas JA, ET, and ROS are relevant to inducing phenylpropanoid metabolism by activating PAL. Moreover, ROS seems to be a key factor in inducing the accumulation of individual phenolic compounds. Our results suggest that their role in activating the primary or secondary metabolism is tissue-dependent, despite the three signaling molecules evaluated being key for the modulation of the wound response. The scientific information generated in this research is needed to envisage strategies to enhance the concentration of antioxidant phenolic compounds in broccoli. Enhancing crop value through wounding stress is attractive to the fresh produce industry in order to generate raw materials with enhanced nutraceutical value that can be used to formulate the next generation of functional foods.

## Figures and Tables

**Table 1:** Effect of diphenyleneiodonium chloride (DPI), phenidone (PHEN), and 1-methylcyclopropene (1-MCP) on the wound-induced accumulation of individual phenolic compounds in broccoli.

Treatment	Individual Phenolic Compounds’ Concentration (mg kg^−1^ DW) ^1, 2, 3^													
	C-glu		β-1-C-glu		α-1-C-glu		4-C-glu		Coumaroyl-QA		5-CQA		3-FQA	
0 h	26.59 ± 4.67	bc	69.88 ± 11.15	cde	105.05 ± 4.35	d	16.91 ± 0.05	f	12.83 ± 1.14	g	76.43 ± 3.62	fg	25.19 ± 0.89	f
0 h-water	20.38 ± 1.58	c	52.19 ± 2.59	e	104.45 ± 9.21	d	16.59 ± 0.41	f	14.65 ± 0.23	fg	74.92 ± 2.58	g	27.64 ± 3.62	ef
21 h	41.20 ± 10.64	b	96.30 ± 19.21	a	175.31 ± 13.15	bc	19.63 ± 0.46	de	67.05 ± 2.42	b	104.11 ± 2.97	bc	51.94 ± 5.05	cd
21 h-water	38.67 ± 10.45	bc	93.42 ± 6.10	ab	145.71 ± 17.59	c	23.19 ± 0.76	bc	76.39 ± 3.97	a	95.33 ± 6.19	cde	101.44 ± 8.43	b
DPI	61.08 ± 10.56	a	61.04 ± 4.07	de	143.33 ± 11.15	c	21.23 ± 1.25	cd	16.12 ± 1.23	fg	76.95 ± 3.57	fg	36.39 ± 1.98	ef
PHEN	31.37 ± 3.79	bc	84.03 ± 3.46	abc	181.41 ± 12.50	b	18.18 ± 0.16	ef	25.79 ± 1.36	e	86.50 ± 3.30	ef	35.69 ± 1.16	ef
1-MCP	22.24 ± 4.20	bc	93.85 ± 4.48	ab	232.88 ± 13.59	a	22.20 ± 0.30	bc	49.41 ± 4.95	c	99.78 ± 3.04	cd	53.26 ± 2.19	c
DPI + PHEN	23.37 ± 1.92	bc	80.10 ± 4.33	abcd	187.65 ± 3.69	b	21.29 ± 0.36	cd	36.90 ± 1.65	d	91.24 ± 3.92	de	274.92 ± 7.45	a
DPI + 1-MCP	25.19 ± 3.91	bc	75.24 ± 3.04	abcd	156.52 ± 10.53	bc	29.72 ± 1.09	a	18.91 ± 0.77	efg	84.10 ± 1.66	efg	40.27 ± 4.51	de
PHEN + 1-MCP	21.79 ± 5.93	c	78.33 ± 0.77	abcd	173.51 ± 1.50	bc	18.50 ± 0.68	ef	19.95 ± 1.51	ef	121.33 ± 4.43	a	33.14 ± 2.11	ef
DPI + PHEN + 1-MCP	36.91 ± 5.57	bc	72.98 ± 3.26	bcde	157.20 ± 12.83	bc	23.29 ± 0.77	b	55.73 ± 2.89	c	114.26 ± 5.66	ab	36.36 ± 2.38	ef
**Treatment**	**Individual phenolic compounds’ concentration (mg kg^−1^ DW)**													
	**4-CQA**		**CA**		**4-SQA**		**4,5-DiCQA**		**4-C-5-FQA**		**1,2-DSG**		**1-S-2-FG**	
0 h	8.37 ± 2.67	d	43.63 ± 1.42	a	15.17 ± 0.17	abcd	30.38 ± 0.85	e	n.d.		n.d.		n.d.	
0 h-water	11.99 ± 3.48	cd	41.84 ± 2.15	a	13.17 ± 0.37	cd	24.40 ± 2.14	e	n.d.		n.d.		n.d.	
21 h	14.45 ± 0.27	bc	23.85 ± 2.15	c	16.18 ± 0.73	ab	52.75 ± 3.03	e	n.d.		n.d.		20.77 ± 0.26	b
21 h-water	15.43 ± 0.65	bc	27.20 ± 1.90	c	16.47 ± 0.87	a	51.01 ± 4.04	e	n.d.		n.d.		16.78 ± 5.55	b
DPI	10.90 ± 2.61	cd	46.35 ± 5.79	a	13.33 ± 0.41	bcd	44.13 ± 6.71	e	n.d.		n.d.		23.48 ± 1.28	b
PHEN	22.85 ± 0.78	a	22.93 ± 0.81	c	12.84 ± 2.23	d	660.30 ± 44.89	c	100.57 ± 14.91	a	n.d.		18.00 ± 1.96	b
1-MCP	10.90 ± 1.00	cd	24.08 ± 0.99	c	15.92 ± 0.47	abc	53.31 ± 2.62	e	n.d.		n.d.		n.d.	
DPI + PHEN	14.09 ± 2.40	bc	26.74 ± 0.27	c	15.08 ± 0.18	abcd	1552.34 ± 53.73	a	n.d.		692.47 ± 11.36	b	2189.58 ± 66.79	a
DPI + 1-MCP	12.40 ± 1.16	cd	46.49 ± 1.72	a	14.45 ± 0.45	abcd	65.97 ± 2.11	e	40.58 ± 0.97	c	23.11 ± 0.06	d	32.94 ± 0.75	b
PHEN + 1-MCP	18.94 ± 1.37	ab	25.47 ± 1.34	c	13.58 ± 2.00	abcd	871.30 ± 58.67	b	71.05 ± 10.26	b	1443.67 ± 10.26	a	10.98 ± 0.78	b
DPI + PHEN + 1-MCP	13.34 ± 1.08	cd	34.38 ± 1.84	b	12.71 ± 0.45	d	447.12 ± 26.18	d	19.39 ± 0.76	d	389.02 ± 12.30	c	23.02 ± 2.64	b
**Treatment**	**Individual phenolic compounds’ concentration (mg kg^−1^ DW)**													
	1,2,2-TSG *			**1,2-DS-2-FG**			**1-S-2,2-DFG**		1,2,2-TSG *			**1,2-DFG**		
0 h	177.91 ± 41.36		a	282.15 ± 62.34		ab	90.39 ± 18.76	ab	15.68 ± 2.13		f	n.d.		
0 h-water	105.55 ± 5.00		b	180.34 ± 8.55		c	58.26 ± 2.04	b	16.94 ± 0.26		ef	n.d.		
21 h	173.44 ± 50.26		a	290.35 ± 8.55		a	94.38 ± 25.00	ab	18.87 ± 0.63		cde	22.07 ± 1.39		ab
21 h-water	140.25 ± 7.44		a	186.35 ± 38.81		bc	81.85 ± 3.85	ab	20.98 ± 0.66		abcd	22.01 ± 1.04		ab
DPI	124.53 ± 9.43		ab	208.26 ± 15.49		abc	88.02 ± 23.75	ab	18.03 ± 0.86		def	16.76 ± 5.72		b
PHEN	158.74 ± 7.00		ab	282.80 ± 13.97		ab	103.06 ± 6.22	a	24.14 ± 0.73		a	22.60 ± 1.45		ab
1-MCP	130.06 ± 7.57		ab	222.39 ± 13.75		abc	70.63 ± 4.60	ab	18.52 ± 0.86		def	22.92 ± 1.50		a
DPI + PHEN	146.89 ± 5.62		ab	264.01 ± 9.82		abc	95.00 ± 5.54	a	23.26 ± 0.39		ab	21.95 ± 1.67		ab
DPI + 1-MCP	144.34 ± 5.64		ab	251.86 ± 8.65		abc	90.80 ± 4.10	ab	22.26 ± 0.40		abc	24.11 ± 0.83		a
PHEN + 1-MCP	161.79 ± 2.33		ab	268.93 ± 4.51		abc	98.66 ± 2.26	a	20.86 ± 2.08		bcd	21.33 ± 1.88		ab
DPI + PHEN + 1-MCP	145.02 ± 5.50		ab	240.60 ± 9.74		abc	85.65 ± 4.18	ab	20.56 ± 0.38		bcd	23.53 ± 0.43		a

^1^ Concentration was determined in broccoli chops 21 h after wounding. ^2^ Concentration is reported as chlorogenic acid equivalents. Data represent the mean of 3 replicates ± standard error of the mean. ^3^ Concentrations were determined based on dry weight. Different letters in the same column indicate statistical difference in the concentration of the compound between treatments according to the LSD test (*p* < 0.05). * Isomeric compounds. Inhibitor concentrations were 317 uM DPI, 10 mM PHEN, and 2000 ppb 1-MCP. Compounds quantified at 280 nm (C-glu; α-1-C-glu; 3-FQA; 4-CQA; CA; 4-SQA; 4,5-diCQA; 4-C-5-FQA; 1,2-DSG; 1-S-2-FG) and at 320 nm (β-1-C-glu; 4-C-glu; Coumaroyl-QA; 5-CQA; 1,2,2-TSG; 1,2-DS-2-FG). * isomeric compounds. Abbreviations: caffeoyl glucose (C-glu); β-1-caffeoyl glucose (β-1-C-glu); α-1-caffeoyl glucose (α-1-C-glu); 4-caffeoyl glucose (4-C-glu); coumaroyl quinic acid (coumaroyl-QA); 5-caffeoylquinic acid (5-CQA); 3-feruloylquinic acid (3-FQA); 4-caffeoylquinic acid (4-CQA); caffeic acid (CA); 4-sinapoylquinic acid (4-SQA); 4,5-dicaffeoylquinic acid (4,5-diCQA); 4-caffeoyl-5-feruloylquinic acid (4-C-5-FQA); 1,2-disinapoylgentiobiose (1,2-DSG); 1-sinapoyl-2-feruloylgentiobiose (1-S-2-FG); 1,2,2-trisinapoylgentiobiose (1,2,2-TSPG); 1,2-disinapoyl-2-feruloylgentiobiose (1,2-DS-2-FG); 1-sinapoyl-2,2-diferuloylgentiobiose (1-S-2,2-diDFG); 1,2,2-trisinapoylgentiobiose (1,2,2-TSG); 1,2-diferuloylgentiobiose (1,2-DFG).

**Table 2:** Primers used in qRT-PCR to evaluate the expression of genes related to the biosynthesis of stress-signaling molecules, primary metabolites, and phenolic compounds in broccoli.

Gene	Description According to GenBank	Forward Primer (5′-3′)	Reverse Primer (5′-3′)	Amplicon Size (bp)
*BoOPR3*	*12-oxophytodienoate reductase 3*	CGATAGGAGCGAGTAAAGTTGG	TTGAGCAAGTCAACCACGGCTA	109
*BoACO4*	*1-aminocyclopropane-1-carboxylate oxidase*	TTGAGGTGATAACCAATGGGAAG	TCCAGGGTTGTAGAATGATGCA	104
*BoPAL1*	*Phenylalanine ammonia-lyase 1*	TGGCAGCAATCTCGACCCTTG	CCATAACTATCGGTGCCTTTGC	124
*BoHQT*	*hydroxycinnamoyl-CoA:quinate hydroxycinnamoyltransferase*	GCTGGGTCAGATTACCAATTTAC	GCTGCCATCATTTGTAGGACTT	125
*BoDAHPS*	*3-deoxy-D-arabino-heptulosonate synthase*	CCGTCAAGCAAGCTTCTCCT	CTCCGGTGTCCATTTGGATT	145
*BoACT2*	*Actin 2*	GTCGCTATTCAAGCTGTTCTCT	GAGAGCTTCTCCTTGATGTCTC	251

Abbreviations: *Brassica oleracea* (Bo).

## Data Availability

The data presented in this study are available within the article.

## References

[1] Plumb G.W., Price K.R., Modes M.J., Williamson G. (1997). Antioxidant properties of the major polyphenolic compounds in broccoli. Free Radical Res..

[2] Cisneros-Zevallos L., Jacobo-Velaázquez D.A. (2020). Controlled abiotic stresses revisited: From homeostasis through hormesis to extreme stresses and the impact on nutraceuticals and quality during pre-and postharvest applications in horticultural crops. J. Agric. Food Chem..

[3] Jacobo-Velázquez D.A. (2022). Definition of biofortification revisited. ACS Food Sci. Technol..

[4] Villarreal-García D., Nair V., Cisneros-Zevallos L., Jacobo-Velázquez D.A. (2016). Plants as biofactories: Postharvest stress-induced accumulation of phenolic compounds and glucosinolates in broccoli subjected to wounding stress and exogenous phytohormones. Front. Plant Sci..

[5] Torres-Contreras A.M., Nair V., Cisneros-Zevallos L., Jacobo-Velázquez D.A. (2017). Stability of bioactive compounds in broccoli as affected by cutting styles and storage time. Molecules.

[6] Guan Y., Hu W., Jiang A., Xu Y., Zhao M., Yu J., Ji Y., Sarengaowa, Yang X., Feng K. (2020). The effect of cutting style on the biosynthesis of phenolics and cellular antioxidant capacity in wounded broccoli. Food Res. Int..

[7] Guan Y., Hu W., Xu Y., Yang X., Ji Y., Feng K., Sarengaowa (2021). Metabolomics and physiological analyses validates previous findings on the mechanism of response to wounding stress of different intensities in broccoli. Food Res. Int..

[8] Guan Y., Hu W., Xu Y., Sarengaowa, Ji Y., Yang X., Feng K. (2021). Proteomic analysis validates previous findings on wounding-responsive plant hormone signaling and primary metabolism contributing to the biosynthesis of secondary metabolites based on metabolomic analysis in harvested broccoli (*Brassica oleracea* L. var. italica). Food Res. Int..

[9] Song C.J., Steinebrunner I., Wang X., Stout S.C., Roux S.J. (2006). Extracellular ATP induces the accumulation of superoxide via NADPH oxidases in Arabidopsis. Plant Physiol..

[10] Jacobo-Velázquez D.A., Martínez-Herández G.B., Rodriguez S.C., Cao C.M., Cisneros-Zevallos L. (2011). Plants as biofactories: Physiological role of reactive oxygen species on the accumulation of phenolic antioxidants in carrot tissue under wounding and hyperoxia stress. J. Agric. Food Chem..

[11] Gastélum-Estrada A., Hurtado-Romero A., Santacruz A., Cisneros-Zevallos L., Jacobo-Velázquez D.A. (2020). Sanitizing after fresh-cutting carrots reduces the wound-induced accumulation of phenolic antioxidants compared to sanitizing before fresh-cutting. J. Sci. Food Agric..

[12] Jacobo-Velázquez D.A., González-Agüero M., Cisneros-Zevallos L. (2015). Cross-talk between signaling pathways: The link between plant secondary metabolite production and wounding stress response. Sci. Rep..

[13] Surjadinata B.B., Jacobo-Velázquez D.A., Cisneros-Zevallos L. (2021). Physiological role of reactive oxygen species, ethylene, and jasmonic acid on UV light induced phenolic biosynthesis in wounded carrot tissue. Postharvest Biol. Technol..

[14] Torres-Contreras A.M., Nair V., Senés-Guerrero C., Pacheco A., González-Agüero M., Ramos-Parra P.A., Cisneros-Zevallos L., Jacobo-Velázquez D.A. (2021). Chemical genetics applied to elucidate the physiological role of stress-signaling molecules on the wound-induced accumulation of glucosinolates in broccoli. Plants.

[15] Blackwell H.E., Zhao Y. (2003). Chemical genetic approaches to plant biology. Plant Physiol..

[16] Foreman J., Demidchik V., Bothwell J.H.F., Mylona P., Miedema H., Torres M.A., Linstead P., Costa S., Brownlee C., Jones J.D.G. (2003). Reactive oxygen species produced by NADPH oxidase regulate plant cell growth. Nature.

[17] Heil M., Greiner S., Meimberg H., Krüger R., Noyer J.-L., Heubl G., Linsenmair E., Boland W. (2004). Evolutionary change from induced to constitutive expression of an indirect plant resistance. Nature.

[18] Watkins C.B. (2006). The use of 1-methylcyclopropene (1-MCP) on fruits and vegetables. Biotechnol. Adv..

[19] Engelberth J. (2011). Selective inhibition of jasmonic acid accumulation by a small α, β-unsaturated carbonyl and phenidone reveals different modes of octadecanoid signalling activation in response to insect elicitors and green leaf volatiles in Zea mays. BMC Res. Notes.

[20] Zhang J., Ma Y., Dong C., Terry L.A., Watkins C.B., Yu Z., Cheng Z.-M. (2020). Meta-analysis of the effects of 1-methylcyclopropene (1-MCP) treatment on climacteric fruit ripening. Hort. Res..

[21] Torres-Contreras A.M., Senés-Guerrero C., Pacheco A., González-Agüero M., Ramos-Parra P.A., Cisneros-Zevallos L., Jacobo-Velázquez D.A. (2018). Genes differentially expressed in broccoli as an early and late response to wounding stress. Postharvest Biol. Technol..

[22] Chotikacharoensuk T., Arteca R.N., Arteca J.M. (2006). Use of differential display for the identification of touch-induced genes from an ethylene-insensitive Arabidopsis mutant and partial characterization of these genes. J. Plant Physiol..

[23] Ruduś I., Sasiak M., Kępczyński J. (2013). Regulation of ethylene biosynthesis at the level of 1-aminocyclopropane-1-carboxylate oxidase (*ACO*) gene. Acta Physiol. Plant..

[24] Thirugnanasambantham K., Durairaj S., Saravanan S., Karikalan K., Muralidaran S., Islam V.I. (2015). Role of ethylene response transcription factor (ERF) and its regulation in response to stress encountered by plants. Plant Mol. Biol. Rep..

[25] Fijimoto S.Y., Ohta M., Usui A., Shinshi H., Ohme-Takagi M. (2000). Arabidopsis ethylene-responsive element binding factors act as transcriptional activators or repressors of GCC box–mediated gene expression. Plant Cell.

[26] Petersen M. (2016). Hydroxycinnamoyltransferases in plant metabolism. Phytochem. Rev..

[27] Rommens C.M., Richael C.M., Yan H., Navarre D.A., Ye J., Krucker M., Swords K. (2008). Engineered native pathways for high kaempferol and caffeoylquinate production in potato. Plant Biotechnol. J..

[28] Niggeweg R., Michael A.J., Martin C. (2004). Engineering plants with increased levels of the antioxidant chlorogenic acid. Nat. Biotechnol..

[29] Vallejo F., Gil-Izquierdo A., Pérez-Vicente A., García-Viguera C. (2004). In vitro gastrointestinal digestion study of broccoli inflorescence phenolic compounds, glucosinolates, and vitamin C. J. Agric. Food Chem..

[30] Li Y., Kong D., He H., Wang H., Wu H. (2019). Correlation of the temporal and spatial expression patterns of HQT with the biosynthesis and accumulation of chlorogenic acid in *Lonicera japonica* flowers. Hort. Res..

[31] Wan C.Y., Wilkins T.A. (1994). A modified hot borate method significantly enhances the yield of high-quality RNA from cotton (*Gossypium hirsutum* L.). Anal. Biochem..

[32] Salzman R.A., Brady J.A., Finlayson S.A., Buchanan C.D., Summer E.J., Sun F., Klein P.E., Klein R.R., Pratt L.H., Cordinnier-Pratt M.-M. (2005). Transcriptional profiling of sorghum induced by methyl jasmonate, salicylic acid, and aminocyclopropane carboxylic acid reveals cooperative regulation and novel gene responses. Plant Physiol..

[33] Livak K.J., Schmittgen T.D. (2001). Analysis of relative gene expression data using real-time quantitative PCR and the 2^−ΔΔ^_Ct_ method. Methods.

